# Comparative Stability of Two Anti-hyperpigmentation Agents: Kojic Acid as a Natural Metabolite and Its Di-Palmitate Ester, Under Oxidative Stress; Application to Pharmaceutical Formulation Design

**DOI:** 10.34172/apb.2022.031

**Published:** 2021-05-30

**Authors:** Sahar Tazesh, Elnaz Tamizi, Mohammadreza Siahi Shadbad, Nazli Mostaghimi, Farnaz Monajjemzadeh

**Affiliations:** ^1^Student Research Committee, Faculty of Pharmacy, Tabriz University of Medical Sciences, Tabriz, Iran.; ^2^Pharmaceutical Analysis Research Center and Faculty of Pharmacy, Tabriz University of Medical Sciences, Tabriz, Iran.; ^3^Department of Pharmaceutical and Food Control, Faculty of Pharmacy, Tabriz University of Medical Sciences, Tabriz, Iran.; ^4^Drug Applied Research Center, Tabriz University of Medical Sciences, Tabriz, Iran.; ^5^Food and Drug Safety Research Center, Tabriz University of Medical Sciences, Tabriz, Iran.

**Keywords:** Stress test, Oxidative stress, Pharmaceutical Preformulation, HPLC, Kinetic, Degradation mechanism

## Abstract

***Purpose:*** Kojic acid (KA) a natural metabolite and its dipalmitate ester, kojic acid dipalmitate(Kadp) are both prescribed to treat skin hyperpigmentation. Stress test reveals the intrinsicstability of active ingredients and leads to selection of the suitable formulations. This researchevaluates the comparative stability of KA and its di-palmitate ester under liquid oxidative stress.

***Methods:*** The HPLC-UV/PDA method with a C_18_ column was utilized. Liquid oxidative stresswas induced using hydrogen peroxide (H_2_O_2_). Degradation was separately induced for eachdrug, and they were compared to each other.

***Results:*** Kadp degraded more rapidly in similar liquid oxidative stress conditions than KA did.The superior degradation model was the first order for both drugs based on the *mean percentage error* (MPE) values, indicating the dependency of the reaction rate on the initial concentrationof the reactive substance. Ring opening was proposed as the most possible theory for KA andKadp oxidative degradation.

***Conclusion:*** It is suggested to use KA instead of Kadp in less stable formulations, such asextemporaneous preparations. The incorporation of antioxidant excipients in Kadp formulationsis recommended for yielding better stability results. Formulating Kadp in the internal phase ofo/w emulsion formulations may protect this susceptive molecule from oxidative degradationduring the shelf life of the pharmaceutical preparation. Further studies are required to study theexact mechanism of the degradation process.

## Introduction

Stability is a crucial factor in manufacturing new formulations in the pharmaceutical market. Pharmaceutical formulators attempt to reach the most stable formulations, which are cost-effective and can gain customer acceptance.^1-3^

**Figure 1:**
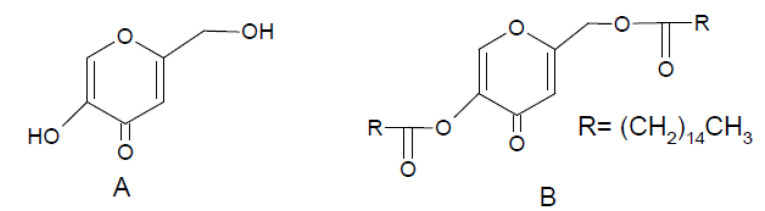
Chemical structure of A) KA and B) Kadp.

Kojic acid (Ka) is a metabolite derived from the activity of various species of *Aspergillus* and *Penicillium* fungi. KA was first used in Japan as a skin lightening agent in 1988.^4^ It acts by inhibiting the activity of the tyrosinase enzyme, which is responsible for the skin hyperpigmentation. Nowadays, it is incorporated as one of the main active ingredients in most cosmetic and skin care products. From a physicochemical viewpoint, KA is classified as a gamma pyrone.^5^ Figure 1 presents the chemical structure of KA and its dipalmitate ester (Kadp).

Esterification of the high water soluble KA with two palmitic acid molecules yields Kadp, which is almost insoluble in water. The ester form of KA is white crystalline powder.^4^

It was previously reported that KA was liable to oxidative damage and should be preserved by antioxidants in topical pharmaceutical formulations. As a hypothesis, it can be assumed that the esterification increases the stability of the main compound by hindering its reactive functional groups.^4^

It has been claimed that the dipalmitate ester possesses higher thermal and photo stability compared to pure KA, and may have more skin absorption owing to its increased lipid solubility,^4^ but this has never been examined or discussed.

In this study, comparative oxidative stability of these two APIs was quantitatively examined, and subsequently the data were fitted to different models to determine the main degradation kinetics in each case separately. According to the literature, there has been no similar study in this area. Stress tests reveal the intrinsic stability of the active ingredients and can lead to the selection of more suitable formulations and improvement of the quality of the marketed products and their shelf life.^1^

Thus, the novelty of this project is to provide comparative stability information for KA and Kadp as anti-hyperpigmentation agents and to propose a possible mechanism of chemical degradation.

## Materials and Methods

### Chemicals

KA (CAS: 501-30-4 ) and KA di palmitate (Kadp) (CAS: 79725-98-7) were purchased from SD Fine Chem, India. Tetrahydrofuran (THF), acetonitrile, methanol, acetic acid, KH_2_PO_4_ and hydrogen peroxide (H_2_O_2_) were all of HPLC grade and prepared from Merck, Germany.

### Differential scanning calorimetry (DSC) analysis

Pure standard samples of KA and Kadp were analyzed using DSC (DSC-60, Shimadzu, Japan) to ensure the melting points. Furthermore, 5-mg samples were heated at a rate of as 15ºC/min from 30ºC to 300ºC in sealed aluminum pans with alumina as a reference material. The TA-60 software (version 1.51) was used for calculations.

### High-performance liquid chromatography (HPLC) method

HPLC equipment was a Knauer high performance liquid chromatography system (Berlin, Germany). The reversed phase column was a high-resolution C18 column (CLIPEUS C18 5 μm, 250 × 2.1 mm, USA) maintained at room temperature (25°C). Injection volume was 25 μL, and the flow rate was set at 0.4 and 0.5 mL/min for KA and Kadp, respectively. The UV spectrum of KA, Kadp and THF in a similar concentration to dilution was recorded using a spectrophotometer (Cecil 7400 series, UK). KA and Kadp were diluted up to 10 µg/mL in the mobile phase of the Kadp HPLC method. THF was also diluted in a similar concentration. PDA analysis was performed using Agilent technologies HPLC system (infinity 1260).

KA was eluted by a mobile phase composed of 99% phosphate buffer pH = 2.5 and 1% methanol.^6^ pH adjustment was performed using orthophosphoric acid.

The mobile phase for detecting Kadp was a mixture of THF (35%) + ACN (30%) + Methanol (29%) + deionized water (5%)+ acetic acid (1%). KA and Kadp were detected by UV absorption at 280 and 250 nm, respectively.^4,6^

### Preparation of standard solutions

KA stock solution (A) was prepared by dissolving 10 mg of the standard powder in 10 mL of deionized water. A total of 10 times dilution of standard (A) resulted in the standard stock solution (B), which was diluted 10 times further to yield stock (C). All calibration KA concentrations (2, 4, 5, 6, 8 and 16 μg/mL) were prepared using stock (C), and the dilution solvent in all steps was deionized water. Each standard was injected 3 times to the HPLC system. In the case of Kadp, THF was used as the primary solvent to prepare standard solutions. Further dilutions were made using acetonitrile. THF is expected to leave the column in the dead volume resulting in a turbulent solvent front which may hide some initial peaks Dilution ratio is considered in all calculations.

First, 10 mg of Kadp standard powders were weighed and dissolved in 10 cc THF to prepare the standard stock solution (A), which was further diluted 10 times using THF to yield stock (B). Standard stock solution (B) was diluted by acetonitrile to prepare 4, 8, 10 and 16 μg/mL of Kadp, which were subjected to the HPLC system. To prepare the calibration curves, each of the prepared concentrations was injected 3 times.

### Stress samples preparations

KA and Kadp were separately examined under liquid oxidative stress conditions. In each stress test, a standard control sample and stress samples were prepared. H_2_O_2_ at a stock concentration of 1.5% was selected to maintain the stress conditions in both drugs. All the experiments were repeated at least 3 times, and the data were presented as the average of the repeated experiments (n = 3).

### Stress test

The highest concentration (16 μg/mL) of the calibration curve was selected for stress testing.

A total of 8 clean glass vials were selected, and stress conditions were established by the following procedure:

- Standard control sample (16 μg/mL): 160 μL of standard B+840 μL of acetonitrile
- Stress samples: 160 μL stock (B) + 420 μL acetonitrile + 420 μL H_2_O_2_ 1.5%

Thus, the final concentration of H_2_O_2_ will reduce to 0.63 %. The sampling intervals of the stress samples of KA were 6, 12, 14, 16, 18, 20, 24, 30, 36, 42 and 48 hours, while for Kadp, the suitable time intervals were 5, 10, 15, 30, 45, 60 and 90 minutes).

Methanol is considered to have a catalytic reaction with peroxide.^7^ To stop the oxidation reactions, at the end of the stress period, 100 and 420 μL of methanol were added to the KA and Kadp stress samples, which were placed in ice bath, respectively. Thus, the final volume of the stress samples was changed, which should be noted in the calculation of real concentrations.

### Partial validation of the analytical HPLC methods

The method transfer was technically performed,^8^ and the HPLC methods for ka and Kadp were fully adopted from the literature. Thus, partial validation of the analytical methods was necessary.^4,6^

For validation, the linearity, accuracy, and limit of detection (LOD) and limit of quantification (LOQ) of analytical HPLC methods were investigated.^9^ To evaluate the linearity of the method, the calibration curve of the concentration to the peak area of KA or Kadp was plotted against the concentrations, and the calibration equation was derived. Three calibration curves were prepared in two consecutive days. Each sample was injected at least 3 times. The LOD and LOQ values were also calculated based on the signal to noise ratio.

## Results

### DSC results

**Figure 2:**
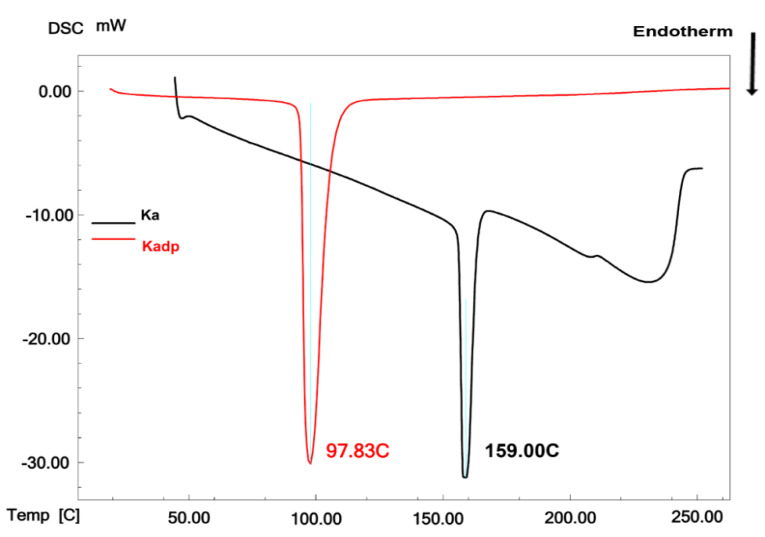
DSC curves of Ka and Kadp.

The melting peak of the pure standard powders of KA and Kadp was determined using the DSC analysis. According to Figure 2, KA and Kadp showed endothermic melting peaks at 159 and 97.83°C, respectively. This is in accordance with the previous literature.^10,11^

### Chromatograms

**Figure 3:**
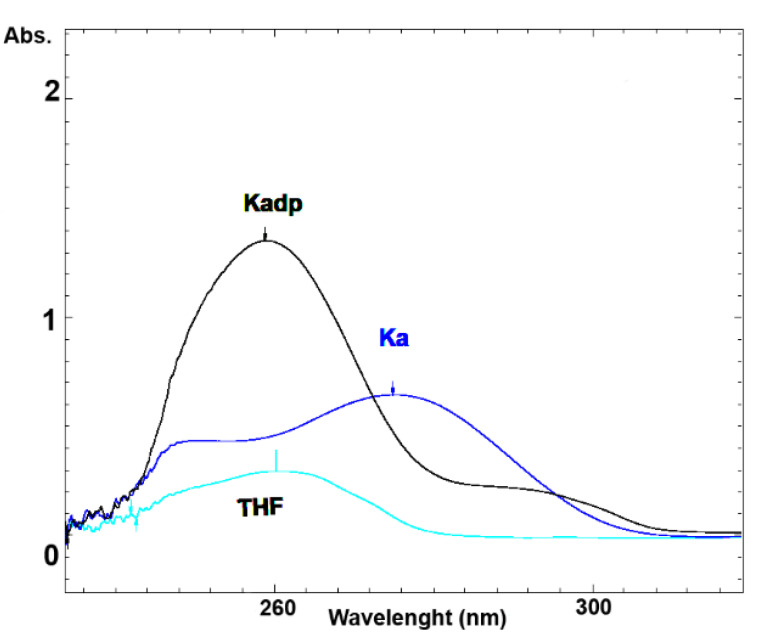
UV spectra of KA and Kadp.

**Figure 4:**
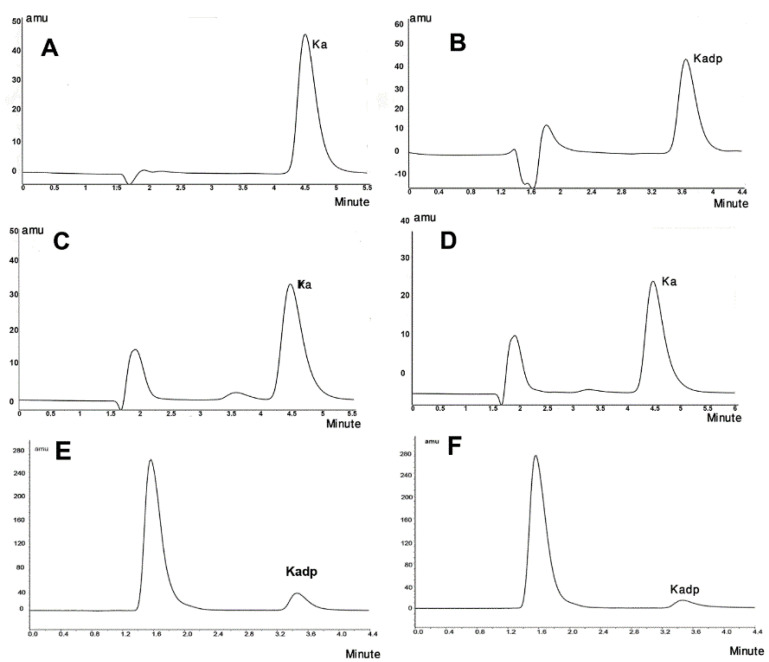
HPLC Chromatograms of (A) KA (8 µg/mL) and (B) Kadp (16µg/mL), (C) KA after 12 h under liquid oxidative stress, (D) KA after 24 hours and 10% degradation (E) Kadp after liquid oxidative stress for 5 minutes and 35% degradation and F) Kadp after 90 minutes and approximately 70% degradation

Figure 3 shows the UV spectra of KA, Kadp and THF diluted in the mobile phase of the Kadp HPLC method. The retention times of KA and Kadp with their individual described chromatographic systems were 4.5 and 3.8 minutes, respectively. Figure 4 (A, B) presents the standard chromatograms of KA and Kadp, respectively.

Figure 4C,D and E,F presents the HPLC chromatograms of some selected stress samples of KA and Kadp, respectively.

### Calibration results

Tables 1 and 2 show the partial validation results for KA and Kadp, respectively.

**Table 1:** Partial validation results for KA

**Concentration** **(µg/mL)**	**Accuracy±SD (%)**	**Inter day** **(RSD %)**	**Intra days** **(RSD %)**
6	98.92 ± 0.03	0.5	1.31
5	99.54 ± 0.02	0.33	2.01
4	99.99 ± 0.05	1.32	1.04

**Table 2:** Partial validation results for Kadp

**Concentration** **(µg/mL)**	**Accuracy±SD (%)**	**Inter day** **(RSD %)**	**Intra days** **(RSD %)**
16	99.94 ± 0.19	1.19	0.12
10	102.82 ± 0.09	0.84	2.23
8	95.05 ± 0.04	0.47	1.17

A linear range of response was seen in the concentration range of 2-8 µg/mL and 4-16µg/mL for KA and Kadp, respectively.^6^ The limits of determination and detection for Ka and Kadp were 0.086 and 0.28 μg/mL, and 1 and 3 μg/mL, respectively.

LOD and LOQ for KA based on the visual signal to noise ratio was calculated to be 0.086 and 0.28 µg/mL, respectively. For Kadp, the LOD and LOQ values were calculated as 1 and 3 µg/mL, respectively.

LOD and LOQ for KA based on visual signal to noise ratio was calculated to be 0.086 and 0.28 µg/mL, respectively. For Kadp the LOD and LOQ values were calculated as 1 and 3 µg/mL, respectively.

### Stress data evaluation

The stability indicating method can quantify API in the presence of degradation products accurately and precisely. Purity of API peaks was checked using a Photo Diode Array detector combined to HPLC. Stress mixtures were injected to HPLC, and the purity factor was calculated for API peaks.

The purity factor greater than 0.980 introduces the stability indicating power of the utilized methods. As described, at certain time intervals, the stress vials were sampled and after suitable dilutions, they were injected into the HPLC system. The remaining amount of the each drug in stressed samples was calculated quantitatively. Data were recorded in each sampling time and fitted to the common kinetic models in solutions. Experiments for model detection were conducted at least 3 times for a constant H_2_O_2_ concentration. Different oxidative agent concentrations may alter the results.

The best kinetic model for the oxidative degradation of the drugs was selected based on mean r-squared (RSQ) and percent error (MPE) of the best line fitted to the data points using the linear regression carried out by the excel software. Tables 3 and 4 show the remaining percentage of KA and Kadp at different time intervals fitted to the common kinetic models (Zero, first, second and third order), respectively.

**Table 3:** Remaining percentage of KA in different time intervals fitted to common kinetic models

	**Zero order**	**First order**	**Second order**	**Third order**
RSQ	0.947	0.965	0.976	0.981
Intercept	14.728	2.694	0.067	0.004
Slope	-0.07	-0.0055	0.0004	0.00006
MPE	1.23	0.4	0.96	1.85

**Table 4:** Remaining percentage of Kadp in different time intervals fitted to common kinetic models

	**Zero order**	**First order**	**Second order**	**Third order**
RSQ	0.587	0.752	0.876	0.935
Intercept	15.984	2.748	0.064	0.005
Slope	-0.131139	-0.011	0.0011	0.0002
MPE	24.03	6.50	11.23	17.49

## Discussion

KA and its dipalmitate ester Kadp are both dermatologically used to treat the skin hyperpigmentation in transdermal pharmaceutical dosage forms. Dermatologists may prescribe these actives as extemporaneous preparations. Up to now, there is no individual or comparative study evaluating the physicochemical stability of these two active agents under liquid oxidative stress conditions. The present study revealed the effect of esterification on the oxidative stability of the KA molecule.

Stress test reveals the intrinsic stability of the active ingredients and can lead to selection of more suitable formulations and to improve the quality of the marketed products and their shelf life. For example, when a drug substance is liable to liquid oxidative stress, a formulator pharmacist may decide to add antioxidants and or avoid some oxidative procedures or metal impurities during the formulation process.

Esterification of pharmaceutical agents may promote the absorption characteristics, increase physico-chemical stability or improve manufacturing parameters such as hygroscopicity and flowability.^12^

Although KA is a water soluble molecule, Kadp is completely lipophilic in nature. Addition of fatty acid chains to the free base of drugs in the transdermal route of administration is regarded to increase the permeability of the resulting agent. Some researchers have reported the increasing effects of adding different fatty acids (lauric acid and myristic acid) on the percutaneous absorption of propranolol.^13^ Stability issues are the other part of the fatty acid addition subject.^14^ Kadp and Phosphonate esters of KA have shown higher efficacy than KA has.^15^

In the oxidative stress test defined by ICH, there is not a clear defined protocol regarding the oxidant type and the length of the exposure. Thus, in developing stress tests, the researcher should specify suitable parameters such as concentration of the oxidant (H_2_O_2_), temperature, sampling time intervals, and suitable stability indicating the HPLC method for quantitative purposes.

Many researchers have used different oxidative conditions using hydrogen peroxide 1-30%.^16-18^ For example, oxidative stress test performed on acyclovir as an antiviral drug was conducted by 1.5% H_2_O_2_ at room temperature, which was performed at 30 minutes up to 3 hours.^16^ Singh et al have provided a comparative list of different approaches.^1^ The selected H_2_O_2_ concentration in the present study was kept at 1.5%. The reaction temperature was also maintained at room temperature, and the reaction mixtures were tested at certain and suitable time intervals, to be able to detect the rate and the kinetic of the oxidative reaction occurred in the reaction solution.

To stop the reaction progress, temperature reduction was performed by placing the reaction mixture samples in the ice bath, followed by methanol addition in equivalent volume to that of the initial H_2_O_2_.

Quantitative evaluations of KA and Kadp were conducted using a reversed phase and non-aqueous reversed phase HPLC separations coupled to the UV/PDA detector, respectively. A simple reverse phase elution was used for KA with a mobile phase consisting of 99% phosphate buffer pH = 2.5 and 1% methanol.^6^ The non-aqueous technique provides the chance of separating high lipophilic compounds using common reverse phase columns as well as common eluents.^19^ In the current study, Kadp as a lipophilic compound was eluted using a mobile phase consisted of THF, acetonitrile, methanol and a small percentage of water equal to 5%.^4^ According to Tables 1 and 2, the partial validation results of the methods were satisfactory.

The maximal UV absorption of Kadp was around 250 nanometers. THF has also very high UV absorption in the wavelength range of 250 nm when diluted in the mobile phase of the Kadp HPLC method (Figure 3). The lipophilic nature of Kadp and its high solubility in THF, along with a high UV cutoff of this solvent, required a special standard preparation procedure.

After stock preparation in THF, several dilutions were made using acetonitrile. A linear range, LOD and LOQ were defined.

The accuracy and precision results for each drug were acceptable. The percentage of the remaining drug at different sampling times was fitted to common reaction models in solutions (zero, one, two and three degrees) to select the best fitting model. Judgments were made based on the resulted RSQ and the produced MPE for each model using the Excel software. Table 3 and 4 show the results for KA and Kadp, respectively. Although the RSQ values propose the third-order kinetics as the best fitted model, the MPE values are less than those of all models for the first-order kinetics. Model selection was based on the least errors.

Kinetic studies allow the researcher to investigate and predict the effects of the concentration of the primary reactants as well as different laboratory conditions on the rate of the reaction. The most common kinetic model for degradation reactions in solutions in pharmaceutical studies is the first-degree model in which the reaction rate depends on the initial concentration of the substance; the equation of this reaction follows the calculation of the natural logarithm of the concentrations.^20^ Furthermore, the first-order reaction model indicates that the degradation reaction is a simple reaction. According to results, the best model for oxidative degradation of KA and Kadp by H_2_O_2_ was determined to be the first-order model. In the pharmaceutical manufacturing, the first-order kinetic indicates that a concentrated product may have lower stability compared to a less concentered one. It should be mentioned that the control sample was stable, and the concentration was kept almost constant during the experiment.

According to the results of data fitting to the first-order kinetic model for KA and Kadp degradation under oxidative stress, KA and Kadp degrade after hours and minutes, respectively. Ultimately, the oxidative degradation of Kadp was determined to be between 5 and 90 minutes, while for KA, the time interval was up to 50 hours. The 30% loss of KA was achieved after 48 hours, while Kadp similar loss (35%) was occurred at about 5 min.

As a chemical viewpoint, KA is a gamma pyrone (Figure 1), and the carbonyl moiety in its structure acts as an ester due to the adjacent vinyl group (aromaticity).^5^ The effect of adding H_2_O_2_ on KA or Kadp was not defined and compared yet.

**Figure 5:**
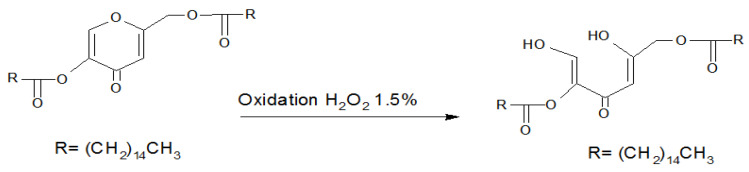
Possible mechanism for the oxidative degradation of Kadp.

The oxidation reaction on the KA molecule may occur in 3 different positions that is one of the branched aliphatic hydroxyl groups and or in the ring heteroatom position. If the first assumption is true, then the fatty acid addition of KA will have the increased stability compared to the parent molecule; however, the reverse will occur if the third position involves the hydrolysis reaction. According to the basic knowledge in the field of heterocyclic compounds, acidic or alkaline reactions may lead to pyrone ring opening.^21,22^ An oxidative process using alkaline hypoiodite is reported, leading to the ring opening and further decomposition to small aliphatic chains.^5^ Based on the results of this project, it is a misunderstanding to believe that hindering hydroxyl groups in KA by two palmitic acid molecules makes it less reactive, since the hydroxyl groups are not the reactive part of the molecule. In the Kadp HPLC method, KA has no retention and washed out the column with the mobile phase (Void volume). As Figure 4 shows, upon Kadp degradation in acidic hydrolytic conditions, the chromatograms exhibited no KA peak. Injecting palmitic acid as a standard solution proved no formation of free palmitic acid upon Kadp Hydrolysis. Initial new peaks may be due to experimental components or degradation products as well as the solvent. More research is needed to exact identification. Previous similar studies on retinyl molecule esterified with palmitate moieties concluded that retinyl palmitate was physico-chemically more labile to photolysis, but it was more resistant to air oxidation than retinol was.^23^ The photostability results obtained by Ihara et al. were in accordance with the findings of the present study for the KA molecule. Figure 5 illustrates the possible theory for the oxidative degradation of Kadp. These findings are of great importance in the preparation of stable pharmaceutical formulations. Ring opening is the possible theory for KA and Kadp degradation under oxidative stress. Given the less stability of Kadp compared to KA, it is logical to conclude that the pyrone ring opening is responsible for the degradation of KA and Kadp. Hydroxyl substitution with an acid functional group as an electron receptor makes the pyrone ring more liable to opening in the oxidative media. Further research is needed to prove this mechanism in detail using mass spectrometry and NMR studies.

## Conclusion

Oxidative stress tests examined the intrinsic stability of molecules and showed which is more stable in the defined condition. In the present study, the stability of KA against oxidative stress was greater than that of dipalmitate ester. Based on our observations, the possible mechanism of ring opening could be proposed in this case.

These outcomes provided a clear understanding of the intrinsic oxidative stability of the studied drug molecules and could lead to the conscious selection of the active ingredient, final dosage form, and excipients substances in the favor of increasing the product’s stability.

It was previously mentioned that both KA and Kadp have skin anti-hyperpigmentation effects, and the superiority of the ester type is owing to its high permeability and thus possibly better efficacy. Therefore, the following recommendations could be considered: It is better to use KA instead of Kadp in less stable formulations, such as extemporaneous preparations. It is also recommended to incorporate antioxidant excipients in Kadp formulations to yield better stability results. Formulating Kadp in the internal phase of o/w emulsion formulations may protect this susceptive molecule from oxidative degradation during the shelf life of the pharmaceutical preparation.

Future studies could take into consideration the stabilization of dipalmitate ester of KA as the more active form of the drug in certain particular formulations. More detailed studies on the mechanism of the drug degradation with accurate analytical tools, LC-MS/MS and NMR for instance, are required in order to investigate the exact mechanism of the degradation process.

## Novelty of the Work

Although both KA and Kadp have been used in hyperpigmentation treatments, no comparative stability information is available for scientists. Additionally, the present work proposes a possible mechanism of chemical degradation.

## Acknowledgments

The authors would like to thank Mrs. Hamideh Najarpour and Ms. Shirin Ahmadi for their contribution in practical oxidative and HPLC method developments, respectively. This paper was extracted from a Pharm. D thesis no. 4016, submitted to the Faculty of Pharmacy, Tabriz University of Medical Sciences and financially supported by a grant from Food and Drug Safety Research Center of the same university (Grant number: 60040).

## Ethical Issues

This study was approved by the ethical committee of Tabriz University of Medical Sciences (code of the present work: IR.TBZMED.REC.1396.45).

## Conflict of Interest

None declared.
